# Supplementary material for: Spatial Variation in Stream-Water Bacterial Communities Across Sites Differing in Wild Chinese Giant Salamander Detection Frequency
Source: Biology (Basel). 2026 Sep 18;15(18):1647. doi: 10.3390/biology15181647 (PMC13604818; doi:10.3390/biology15181647)
Supplement: Supplementary file 1 [file biology-15-01647-s001.zip › Data Related to “Spatial Variation in Stream-Water Bacterial Communities across Sites Differing in Wild Chinese Giant Salamander Detection Frequency”/Microbial Data/result/02.FeatureAnalysis/featureTable_qzv/sample-frequencies.pdf]

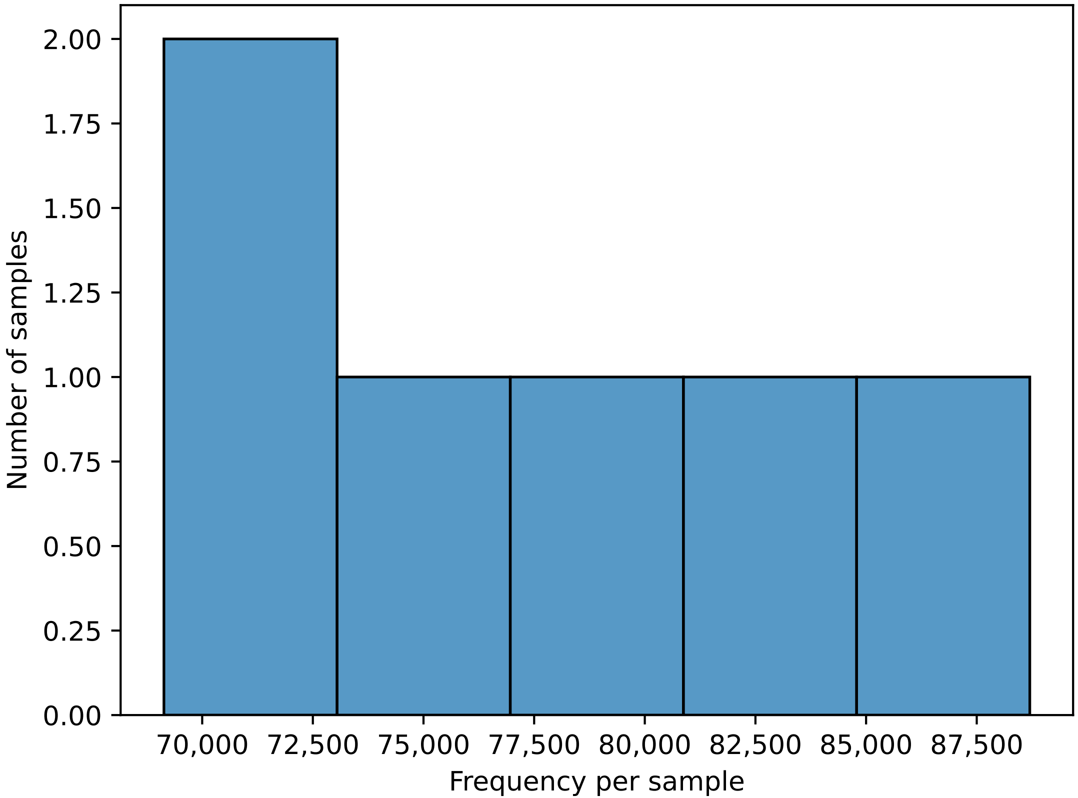
2.00
1.75
1.50
1.25
1.00
0.75
0.50
0.25
0.00
Number of samples
70,000
72,500
75,000
77,500
80,000
82,500
85,000
87,500
Frequency per sample
